# A Rare Case of Actinomycosis in a Patient With Systemic Lupus Erythematosus

**DOI:** 10.7759/cureus.93432

**Published:** 2025-09-28

**Authors:** Vinaya Gopalaswamy, Sarit S Pattanaik, Manoj K Parida, Saumya R Tripathy, Bidyut K Das

**Affiliations:** 1 Clinical Immunology and Rheumatology, Srirama Chandra Bhanja Medical College and Hospital (SBCMCH), Cuttack, IND; 2 Rheumatology, Srirama Chandra Bhanja Medical College and Hospital (SBCMCH), Cuttack, IND

**Keywords:** immunosuppression, primary cutaneous actinomycosis, prolonged antibiotic therapy, surgical and medical therapy, systemic lupus erythematosus

## Abstract

Actinomycosis is a rare, chronic infection caused by Actinomyces species, which are normal commensals of human mucosa. It commonly involves the orocervicofacial, thoracic, abdominal, and pelvic regions. It can also affect other sites, such as cutaneous tissue, as seen in our case. We report a case of a 35-year-old woman with systemic lupus erythematosus (SLE) and lupus nephritis on immunosuppressive therapy who developed a large, nodular cutaneous actinomycosis lesion over the right calf. A biopsy confirmed Actinomyces, and she responded to a six-month course of amoxicillin-clavulanate. However, the lesion recurred a year later, requiring surgical excision due to persistent disease despite prolonged antibiotic therapy. Over a two-year follow-up, no further recurrence was noted. This case highlights the diagnostic challenges of cutaneous actinomycosis, particularly in SLE patients, and emphasizes the role of histopathological confirmation, prolonged antibiotic therapy, and surgical intervention when necessary.

## Introduction

Actinomycosis is a rare, chronic, granulomatous infection caused by gram-positive, filamentous Actinomyces species [1]. These bacteria are normal commensals of the human mucosa and typically become pathogenic following a breach in the mucosal barrier [1]. Actinomyces primarily affect the orocervicofacial, thoracic, abdominal, and pelvic regions [2]. However, it can also involve other sites, such as cutaneous and subcutaneous tissue.

Systemic lupus erythematosus (SLE) is an autoimmune disease characterized by multi-organ involvement and immune system dysregulation. Patients with SLE, especially those undergoing immunosuppressive therapy, are at an increased risk of infections, including bacterial, fungal, and opportunistic infections [3]. Although actinomycosis is not classically considered an opportunistic infection, increasing reports suggest that immunocompromised individuals like patients on steroids [2], may have a predisposition to developing Actinomyces infections. Here, we present a unique case of cutaneous actinomycosis in an SLE patient with lupus nephritis, highlighting diagnostic challenges, treatment responses, and a review of similar reported cases.

## Case presentation

This report is of a 35-year-old female diagnosed with SLE for the past 12 years with mucocutaneous and nephritis as the affected domains. She received induction therapy with three doses of pulse methylprednisolone and intravenous (IV) cyclophosphamide (Euro-Lupus regimen) in 2017 for nephritis, followed by maintenance therapy with mycophenolate (2 g/day), prednisolone (2.5 mg/day), and hydroxychloroquine (200 mg/day).

**Figure 1:**
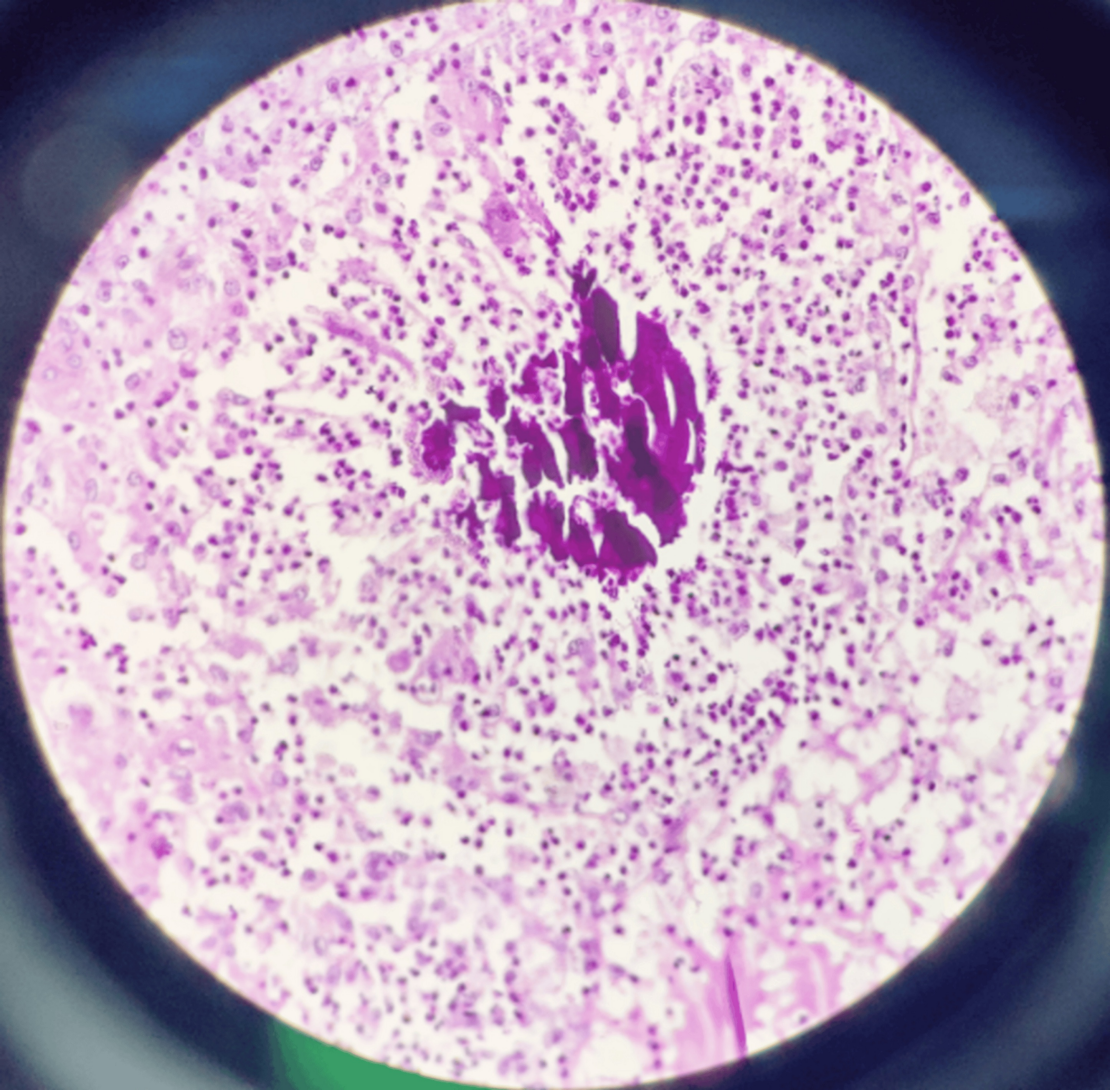
Clumps of basophilic bacteria with branching filaments surrounded by eosinophilic club-shaped ends (Splendore Hoeppli reaction) surrounded by dense acute inflammatory infiltrate with necrotic areas.

In April 2021, she presented with a large nodular mass lesion over the right calf for two months which was progressively increasing in size and non-painful. The lesion was not fixed to the underlying muscle. There was no discharge or surface changes. She had no history of fever or lymphadenopathy. As the patient was immunosuppressed, the differential diagnosis of hypertrophic variant of lupus vulgaris, actinomycosis, sporotrichosis, and fungal infection were considered. Her routine labs were unremarkable. Ultrasonography showed a hypoechoic, nodular, conglomerated, elongated lesion measuring 11 × 7 cm in the subcutaneous plane in the posterior aspect of the right mid-calf with minimal internal flowing debris with normal underlying muscle. Punch biopsy of the mass showed infiltration of polymorphs and actinomycotic colony formation in the epidermis and dermis with Splendore Hoeppli formation (Figure 1). She was given amoxycillin-clavulanate for six months with good response.

**Figure 2:**
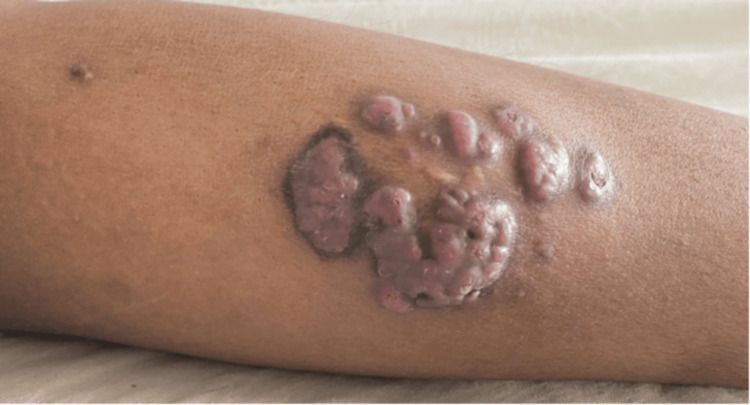
Clinical image of the lesion during recurrence in April 2022

In April 2022, she presented with recurrence of a nodular variegated mass with satellite lesions in the same region (Figure 2) and repeat biopsy showed non-specific granulomatous tissue with foci of ill-defined suppurative granuloma and foreign body-type giant cells and positive staining for Actinomyces. Simultaneously, the patient had mucocutaneous and arthritis flares that were managed by increasing the steroid dose. In view of the frequent flares of SLE that required disease-modifying antirheumatic drugs (DMARDs), excision of the lesion was performed. In the follow-up at two years, the patient had not shown any further recurrence.

## Discussion

Actinomycosis is an uncommon, endogenous infection affecting all age groups caused by Actinomyces species [1]. Actinomyces is a gram-positive, filamentous, non-acid-fast, anaerobic to microaerophilic bacteria that normally resides on the mucosal surface of the mouth, colon, and genitourinary tract. Classically, it is an indolent, slowly progressive granulomatous disease that mimics malignancy, as it does not respect tissue boundaries [4]. 

Mucosal disruption can lead to infection, most commonly orocervicofacial, abdominal, thoracic and pelvic region but virtually any site can be involved [1]. Our case was atypical in its location involving skin and subcutaneous tissue of the lower extremities. There are previous reports of lower extremity disease among intravenous drug users due to infection from of an injection needle, following foot trauma from a toothpick puncture. No such antecedent trauma was identified in our patient.

The disease can occur in both immunocompetent and immunocompromised individuals [2]. Infections are common in individuals who do not seek or have access to health care or an intrauterine device (IUD) in place for a prolonged duration or who receive bisphosphonates [5], hence has not been considered an opportunistic infection. Reports have shown individuals with HIV, transplantation, common variable immunodeficiency (CVID), chronic granulomatous disease (CGD) [6], or on treatment with tumor necrosis factor (TNF) inhibitors [7], steroids, bisphosphonates [5], radiotherapy [8], and chemotherapy are susceptible to actinomyces infection.

Of the six documented cases (Table 1) [9-14], four occurred in females, with a collective age range of 27 to 49 years. A prominent feature was the high prevalence of renal disease (five out of six), and had end-stage renal disease (ESRD), including two post-transplant recipients, and most are on some form of immunosuppressive treatment. The clinical presentations of actinomycosis were diverse, involving the oesophagus (two cases), larynx (two cases), neck, and pelvis. An identifiable precipitating factor either prior to intubation [9,10] or an IUD [11] was noted, though a significant latency period of over one year preceded diagnosis [9,11]. This extended timeframe complicates a direct temporal association but remains consistent with the characteristically insidious progression of actinomycotic infections.

**Table 1:** Review of case reports of SLE with actinomycosis. ESRD: end-stage renal disease, IUCD: intrauterine contraceptive device, LN: lupus nephritis, SLE: systemic lupus erythematosus.

Patient details	Background disease	Immunosuppression	Phenotype	Precipitating event	Treatment	reference
35/F	SLE with ESRD	Details not specified	Largyneal actinomycosis	Intubation 1 year back	Excision followed by oral penicillin for 3 months	[9]
47/M	SLE with ESRD post-transplant	NA	Laryngeal	Intubation recent past	Penicillin for 3 months	[10]
49/F	SLE with Class IV LN	Steroids and cyclophosphamide followed by azathioprine	Pelvic actinomycosis	IUCD 2 years back	Excision followed by ampicillin IV for 1 month followed by oral antibiotic	[11]
28/F	SLE with ESRD on haemodialysis	Details not specified	Oesophageal actinomycosis	NA	IV penicillin for 1 week followed by ampicillin for 6 months	[12]
27/M	SLE with ESRD post-cadaveric transplantation	Prednisolone praquenil prograf	Oesophageal actinomycosis	NA	IV penicillin for 4 weeks followed by oral antibiotic	[13]
34/F	SLE	Methotrexate hydroxychloroquine	Cervical	NA	Amoxicillin for 12 months	[14]

Routine blood investigations are nonspecific and may show anemia, mild leukocytosis, and elevated erythrocyte sedimentation rate (ESR) and C-reactive protein (CRP). Imaging in early stages may show nonspecific inflammatory changes with abscess formation but no lymphadenopathy. At later stages, it may demonstrate tissue infiltration mimicking malignancy, with sinus formation, which, although characteristic, is not specific to actinomycosis [2].

Diagnosis is primarily based on histopathology, which identifies sulfur granules and demonstrates gram-positive filamentous bacteria [2]. Sulfur granules, though not specific to actinomycosis, appear as round or oval basophilic masses (colonies of organisms) with eosinophilic terminal clubs. Microbiologic isolation confirms the diagnosis, but the isolation rate is less than 50% [6].

Treatment requires prolonged treatment with high doses of antibiotics (penicillin, amoxicillin, erythromycin, tetracyclines, doxycycline, and clindamycin) [2]. Combined medical-surgical therapy is advocated in selected cases. Our patient had recurrent lesions on her calf, without any obvious trauma or inciting event and didn’t respond to prolonged high doses of therapy requiring complete surgical excision. Although rare, diagnosis can be challenging, especially in atypical sites or without a history of trauma. Correct diagnosis is imperative to determine the appropriate antibiotic duration and the need for surgical excision.

## Conclusions

This case highlights a cutaneous presentation of actinomycosis in a patient with SLE on immunosuppressive therapy. The unusual site of infection, absence of trauma, and recurrence despite prolonged antibiotics made diagnosis and management challenging. Histopathology was key to diagnosis, and surgical excision was required due to incomplete response to medical therapy. A combined approach of antibiotics and surgery may be needed in resistant or recurrent cases.

## References

[1] Könönen E, Wade WG (2015). Actinomyces and related organisms in human infections. Clin Microbiol Rev.

[2] Wong VK, Turmezei TD, Weston VC (2011). Actinomycosis. BMJ.

[3] Barber MR, Clarke AE (2020). Systemic lupus erythematosus and risk of infection. Expert Rev Clin Immunol.

[4] Russo TA (2022). Actinomycosis. Harrison’s Principles of Internal Medicine. 21st ed.

[5] Schipmann S, Metzler P, Rössle M (2013). Osteopathology associated with bone resorption inhibitors - which role does Actinomyces play? A presentation of 51 cases with systematic review of the literature. J Oral Pathol Med.

[6] Reichenbach J, Lopatin U, Mahlaoui N (2009). Actinomyces in chronic granulomatous disease: an emerging and unanticipated pathogen. Clin Infect Dis.

[7] Breton AL, Lamblin G, Pariset C, Jullien D (2014). Cutaneous actinomycosis associated with anti-TNF-alpha therapy: report of two cases. Dermatology.

[8] Hansen T, Kunkel M, Kirkpatrick CJ, Weber A (2006). Actinomyces in infected osteoradionecrosis--underestimated?. Hum Pathol.

[9] Abed T, Ahmed J, O'Shea N, Payne S, Watters GW (2013). Primary laryngeal actinomycosis in an immunosuppressed woman: a case report. Ear Nose Throat J.

[10] Sims HS, Heywood BB (2007). Post-transplant actinomycosis of the posterior glottis involving both vocal processes. Otolaryngol Head Neck Surg.

[11] Oztekin K, Akercan F, Yucebilgin MS (2004). Pelvic actinomycosis in a postmenopausal patient with systemic lupus erythematosus mimicking ovarian malignancy: case report and review of the literature. Clin Exp Obstet Gynecol.

[12] Nagaraju SP, Kirpalani DA, Bhabhe AS, Prasad R, Shah H, Kirpalani AL (2014). Esophageal actinomycosis in a patient with end-stage renal disease. Hemodial Int.

[13] Welling RD, Cardona DM, Thompson WM (2009). Esophageal actinomycosis: a case report and review of radiographic findings. J Radiol Case Rep.

[14] Sweis RF, Propes MJ, Hyjek E (2011). Actinomyces-induced inflammatory pseudotumor of the lymph node mimicking scrofula. Ann Intern Med.

